# PC Deficiency Testing: Thrombin-Thrombomodulin as PC Activator and Aptamer-Based Enzyme Capturing Increase Diagnostic Accuracy

**DOI:** 10.3389/fcvm.2021.755281

**Published:** 2021-10-11

**Authors:** Sara Reda, Heiko Rühl, Jana Witkowski, Jens Müller, Anna Pavlova, Johannes Oldenburg, Bernd Pötzsch

**Affiliations:** Institute of Experimental Hematology and Transfusion Medicine, University Hospital Bonn, Bonn, Germany

**Keywords:** functional protein C assays, thrombin-thrombomodulin complex, thrombophilia testing, aptamer, diagnostic sensitivity

## Abstract

Protein C (PC) activity tests are routinely performed in a thrombophilia workup to screen for PC deficiency. Currently used tests combine conversion of PC to activated PC (APC) by the snake venom Protac with subsequent APC detection through hydrolysis of a chromogenic peptide substrate or prolongation of a clotting time. In this prospective cohort study, we analyzed how different modes of PC activation and subsequent APC determination influence the diagnostic accuracy of PC activity testing in a cohort of 31 patients with genetically confirmed PC deficiency. In addition to chromogenic and clot-based measurement, an oligonucleotide-based enzyme capture assay utilizing a basic exosite-targeting aptamer was used for APC detection. To study the influence of the PC activation step on diagnostic sensitivity, PC activation through Protac and through the thrombin-thrombomodulin (TM) complex were compared. Twenty-six (84%) and 24 (77%) PC deficient patients were identified as true-positive using the chromogenic and the clot-based PC activity assay, respectively. True-positive results increased to 27 (87%) when the basic exosite-targeting aptamer approach was used for APC measurement. Additional replacement of the PC activator Protac by thrombin-TM gave true-positive results in all patients. These data indicate that the mode of PC activation is crucial in determining the accuracy of PC activity testing and that diagnostic sensitivity can be significantly improved by replacing the PC activator Protac with thrombin-TM. APC detection using a basic exosite-targeting aptamer achieves high sensitivity toward mutations outside the active center while being less subject to interfering factors than clot-based PC activity assays.

## Introduction

Protein C (PC) is the zymogen of the serine protease activated PC (APC) and circulates in plasma at a concentration of ~70 nM (1). The two-chain PC molecule has a multidomain structure comprising a N-terminal γ-carboxyglutamic acid domain, two epidermal growth factor-like domains and a C-terminal serine protease domain (2). PC becomes converted into APC by an activation complex formed between thrombin and thrombomodulin (TM) on the surface of endothelial cells. The activation efficacy is accelerated by binding of PC to the endothelial protein C receptor (EPCR) (3). Once formed, APC down-regulates thrombin formation by proteolytic cleavage of the activated cofactors V (FVa) and VIII (FVIIIa) and induces cytoprotective signaling through protease activated receptor activation (4, 5). The anticoagulant functions of APC are augmented by complex formation with its cofactor protein S (PS) (3).

Inherited PC deficiency is associated with a life-long increased risk of venous thromboembolism and microvascular thrombosis depending on the residual PC activity. In severe forms of PC deficiency, such as in homozygous or compound heterozygous forms, purpura fulminans dominates the clinical phenotype (6). Purpura fulminans is a thrombo-inflammatory disease affecting the microcirculation and leading to skin necrosis and organ failure. In milder forms of PC deficiency venous thrombosis dominates the clinical phenotype as characterized by an ~15-fold increased risk for venous thromboembolism (VTE) (7, 8).

Laboratory tests for PC deficiency include activity testing, antigen testing, and genetic analysis. The activity assays combine conversion of PC to APC in patient plasma with subsequent APC detection either through prolongation of a clotting time or hydrolysis rates of a chromogenic peptide substrate. The prolongation of the clotting time relies on APC dependent inactivation of FVIIIa and FVa and is measured using the activated partial thromboplastin time (aPTT) or the Russell viper venom time. All commercially available PC activity assays use Protac for APC generation. Protac is a glycosylated single-chain serine protease isolated from the venom of Agkistrodon contortrix, that converts PC to APC by rapidly hydrolyzing the Arg169-Leu170 bond of the activation peptide independently of thrombin or TM (9, 10). PC antigen can be measured by antibody-based assays.

Protac-based chromogenic assays are simple, precise and easy to handle, and therefore recommended by current guidelines on laboratory testing for PC deficiency (11, 12). The diagnostic sensitivity of this type of assay, however, might be limited by two factors. First, the physiological activation complex is replaced by Protac, and, second, the macromolecular substrates FVa and FVIIIa are substituted by a peptide substrate. Unlike FVa and FVIIIa, cleavage of the peptide substrate does not involve molecular regions of APC outside the catalytic triad such as the basic exosite, which consists of several basic residues clustered on the 39 and 70–80 loops. Hence, mutations located within these structures cannot be detected through cleavage of the peptide substrate, although they seem to be rare (13, 14). Clotting-based assays will be able to identify these types of mutations. However, clotting-based assays exhibit interference with several other factors including increased levels of FVIII, the FV Leiden mutation, antiphospholipid antibodies, and the intake of direct oral anticoagulants. These frequently occurring confounding factors together with the relatively high inter- and intra-laboratory variation are main reasons that most laboratories prefer the chromogenic test as the screening assay (12).

Although genetic testing can be considered the gold standard for identification of PC deficiency, it is not an appropriate screening assay. This is because of the high diversity and heterogeneity of PC deficiency with more than 270 mutations in the PC gene (PROC) identified so far (15). Moreover, there is no clear correlation between the clinical phenotype and the underlying genetic defect (16, 17). Because of these reasons PC activity testing remains the method of choice for PC deficiency screening.

With the DNA aptamer HS02-52G we have recently identified a ligand that binds with high affinity and selectivity to the basic exosite of human APC (18). Since the catalytic center of APC is not inhibited by HS02-52G binding, this aptamer was successfully used to construct an oligonucleotide-based enzyme capture assay (OECA) (19). In this APC-OECA APC is immobilized to HS02-52G and subsequently quantified through cleavage rates of a fluorogenic peptide substrate. Since binding of APC to HS02-52G critically involves the basic exosite of the enzyme, the APC-OECA should be sensitive to mutations that affect the basic exosite without influencing the active center of APC. To test this hypothesis, we combined Protac-induced PC activation with subsequent APC detection by OECA. Results obtained by this PC-Protac-OECA in a cohort of 31 patients with different types of genetically confirmed PC deficiencies were compared with the results obtained using a chromogenic PC activity test, an aPTT-based assay, and PC antigen testing.

In addition, different modes of PC activation, either by Protac or by thrombin-TM complexes might influence the sensitivity of PC activity testing. While the molecular regions of the PC molecule, that are required for binding of PC to thrombin-TM, have been identified, the molecular regions involving Protac binding have not been identified so far. If these binding regions are different from the binding regions involved in thrombin-TM binding, the PC activity may be overestimated. To investigate the influence of the type of APC generation on PC activity testing, we comparatively analyzed Protac- and thrombin-TM-based APC generation rates in healthy individuals and patients with PC mutations. The results obtained suggest that the sensitivity of PC deficiency testing might be improved when using thrombin-TM as PC activator. Improving the structure function analysis in hereditary PC deficiency may help to better estimate the prothrombotic potential and guide clinical decisions in the diagnosis, evaluation, and management of patients with PC deficiency in the future.

## Materials and Methods

### Reagents and Materials

Protac and the fluorogenic peptide substrate Pefafluor PCa (Pyroglu-Pro-Arg-AMC) were obtained from Pentapharm (Basel, Switzerland). Human TM and the peptide Gly-Pro-Arg-Pro were purchased from Sigma-Aldrich (Saint Louis, USA). Human α-thrombin was from Haematologic Technologies (Essex Junction, USA) and obtained from CellSystems (St. Katharinen, Germany). Aprotinin was purchased from PanReac AppliChem ITW Reagents (Darmstadt, Germany). Bivalirudin (Angiox^®^) was obtained from The Medicines Company (Oxfordshire, UK). The biotinylated APC-binding aptamer HS02-52G was synthesized and purified by Microsynth (Balgach, Switzerland).

### Study Participants and Blood Sampling

This prospective study was conducted from March to September 2018 at the Institute of Experimental Hematology and Transfusion Medicine, Bonn, Germany. Study participants were healthy individuals and patients with PROC gene mutations, who were recruited from our blood donation service and the thrombophilia outpatient clinic of our institution, respectively. Inclusion criteria for the healthy controls were eligibility for blood donation. Inclusion criteria for the patients were a PROC gene mutation associated with reduced PC activity, both diagnosed in our laboratory at an earlier visit to our clinic. Patients with a history of VTE were included only, if the VTE had occurred at least 6 months ago, allowing for short-time interruption of anticoagulant treatment. Exclusion criteria for all study participants consisted of treatment with vitamin K antagonists within 2 weeks preceding blood sampling, other anticoagulant drugs within 72 h preceding blood sampling, acute or chronic infections, and, for female participants, pregnancy and breast feeding. Blood donors visiting our blood donation service were recruited consecutively within 1 day, until a cohort of 10 male and 10 female individuals was reached. Patients with PROC gene mutations were recruited at or prior to scheduled routine visits to our clinic, reaching a cohort of 31 recruited patients over the course of the study. Blood was drawn from an antecubital vein into trisodium citrate (10.5 mmol, final concentration) tubes (Sarstedt, Nümbrecht, Germany) after discarding the first 2 ml. Plasma samples were prepared by centrifugation (2,600 × g, 10 min) within 60 min after blood draw and stored at < -70°C until used. Collection of blood samples was completed in all recruited study participants, and all collected blood samples were analyzed and reported.

### Genetic Analysis

Genetic analysis was performed before the patients were part of this study. Genomic DNA was isolated from peripheral whole blood by a standard salting-out procedure (20). The DNA concentration was determined and standardized to 100 ng/μl. All exons and flanking intron region sequences for the PROC gene were determined by direct sequencing. DNA sequencing was performed on both forward and reverse strands using the BigDye Terminator Cycle sequencing V1.1 Ready Reaction kit and an automated 3130xl Genetic Analyzer (Applied Biosystems, Foster City, CA, USA). Final sequence reading and mutation documentation were performed using the Sequence Analysis software package (Applied Biosystems). Sequences were analyzed using Applied Biosystems SeqScape Software version 2.5. Multiplex ligation-dependent probe amplification (MLPA) was performed according to the manufacturer's recommendations using the SALSA MLPA KIT P265 (MRC-Holland, Amsterdam, Netherlands) for all samples for which no causative point mutation was identified. The ligation products and controls were amplified by PCR using 100 ng of genomic DNA. The amplification products were run an ABI PRISM 3130XL DNA Sequencer with the GeneScan 400 LIZ size standard (Applied Biosystems) and analyzed by GeneMapper Software 5.0 (Applied Biosystems).

### PC Activity and PC Antigen Testing Using Commercially Available Assays

Chromogenic PC activity (PC-chrom) and clotting-based PC activity (PC-coag) were measured using the Atellica COAG 360 coagulation analyzer (Siemens Healthcare Diagnostics, Eschborn, Germany) and Berichrom^®^ Protein C and Protein C (coagulometric) reagents, respectively (Siemens Healthcare Diagnostics Products, Marburg, Germany). Both assays utilize Protac-based reagents to activate PC. The PC-chrom assay then uses a chromogenic substrate for quantitative determination of functionally active PC, while the coagulometric assay measures the prolongation of aPTT. Reference ranges provided by the manufacturer were 63–143% for the coagulometric PC activity assay, and 68–150% for the chromogenic PC activity assay. PC antigen levels were determined using an enzyme-linked fluorescence assay (Vidas^®^ PC test, bioMérieux, Marcy-l'Etoile, France) with a reference range of 65–140%.

### PC-OECA Activity Testing

The PC-OECA activity test is a two-stage assay that combines a PC activation step with subsequent quantification of APC using the APC-OECA. For PC activation plasma samples were diluted 1:10 in activation buffer and the PC activators Protac (0.6 U/ml final concentration) or thrombin-TM added. The activation buffer (10 mmol/l Tris-buffered saline) contained 0.1% BSA and 10 μmol/l (final concentration) of the reversible APC inhibitor aprotinin to prevent inactivation of formed APC by endogenous APC inhibitors prior to measurement. After incubation at room temperature (RT) for 30 min APC was quantified using the APC-OECA essentially as described by Müller et al. (19). In brief, Maxisorp Fluoronunc microtiter modules (Nunc A/S, Roskilde, Denmark) were initially coated with 10 μg/mL of BSA-biotin (100 μL/well). After incubation at 4°C overnight, wells were washed, and a solution of 10 μg/mL streptavidin added and incubated for 1 h at RT, and emptied plates stored at −20°C until used. For running the OECA, the 3'-biotinylated aptamers HS02-52G were placed in the designated wells. After washing, the plasma samples, in which APC formation had been induced by Protac or thrombin-TM, were transferred to the aptamer-coated wells. After incubation and washing, the fluorogenic APC peptide substrate Pyroglu-Pro-Arg-AMC was added to the wells for detection of captured APC. Changes in fluorescence over time were measured using a plate fluorescence reader (Synergy 2, BioTek Instruments, Bad Friedrichshall, Germany). Calibrators consisted of a dilution series of pooled citrated plasma in PC deficient plasma (Siemens Healthcare Diagnostics Products, Marburg, Germany) that covered a range from 0 to 140% chromogenic PC activity. Data obtained from the calibrators were interpolated by 4-parameter curve fit and used to calculate the PC concentration. All samples were assayed in triplicate.

### Data Analysis

All authors had access to primary data. Normality of data was tested using the Shapiro-Wilk test.

The agreement between the different assays was assessed using Pearson correlation and Bland-Altman analysis. All calculations were performed using the XLSTAT statistical and data analysis solution software (Addinsoft, Boston, MA, USA).

## Results

### Study Population

**Figure 1:**
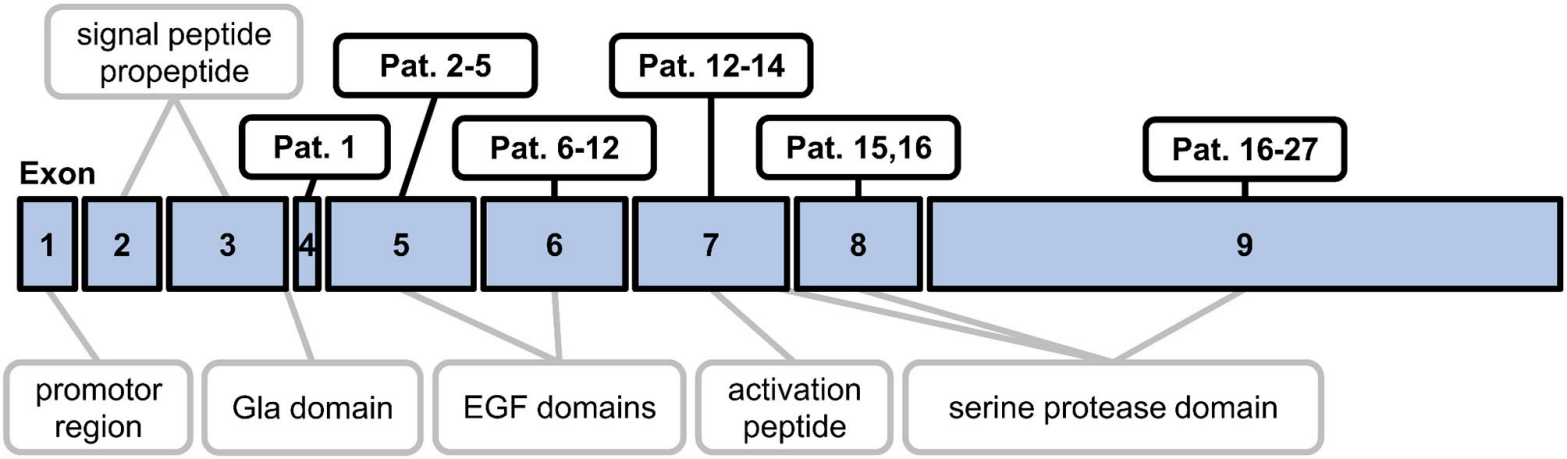
Schematic map outlining the exon mutations in the study population. The diagram shows the nine exons of the PROC gene and the corresponding regions of preproprotein C. EGF, epidermal growth factor.

The study population consisted of 31 patients (25 females, mean age 38.6, range 13–72 years) with genetically confirmed heterozygous PC deficiency and of 20 healthy individuals (10 females) with a mean age of 35.5 years (range 21–58 years). A comparison of male vs. female healthy individuals did not show any significant differences (Student's *t*-test *P* > 0.05) regarding age (34.1 vs. 36.1), PC activity in the PC-chrom assay (116 vs. 114%) and the PC-coag assay (102 vs. 103%), and PC antigen levels (80% each). Within the cohort of PC deficiency patients, 14 patients (45%) had a history of deep vein thrombosis, thereof six (19%) with a history of recurrent deep vein thrombosis and five (16%) with consecutive pulmonary embolism. Thrombophilia screening in the remaining 17 (55%) PC deficient patients without a history of thrombosis was initiated because of pregnancy complications or a positive family history of thrombosis or PC deficiency. At time of analysis no patient was under treatment with heparin or an oral anticoagulant. Within the cohort of PC deficiency patients 25 different mutations (7 novel mutations) had been identified before PC activity and antigen testing were performed as part of this study. The spectrum included 15 (61%) missense mutations, five (16%) nonsense mutations, three (10%) splice-site mutations, two (6%) nucleotide variations, and two (6%) large deletions (Supplementary Table 1). The positions of the mutations in the exons of the PROC gene are shown in Figure 1.

### Diagnostic Sensitivities of the Routine PC Assays

The PC-chrom assay showed PC activities within the reference range in 5 patients indicating a diagnostic sensitivity of 84% (Table 1). Two of the patients classified as false-negative showed a history of VTE. The PC-coag assay gave false-negative results in 7 patients (Table 1) with 3 of them showing a history of VTE. Four patients with three different mutations were recognized as false-negative in both routine PC activity assays.

**Table 1:** Results of routine PC deficiency testing in patients with PC gene mutations (*n* = 31).

**Type of assay**	**Patients tested true-positive, *n***	**Patients tested false-negative, *n***
PC-chrom	26	5
PC-coag	24	7
PC antigen	29	2

*PC, protein C; PC-coag, clot-based PC activity assay; PC-chrom, chromogenic PC activity assay*.

PC antigen levels below the normal range were observed in all but two patients showing the identical splice-site mutation indicating a sensitivity of 94% (Table 1). The PC activity/antigen ratio was >1.0 based on the PC-chrom assay and >0.8 based on the PC-coag assay in all patients characterizing them as type 1 PC deficiency (21, 22). All PC activity and antigen results are listed in Supplementary Table 1.

### Exosite-Specific APC Detection Increases the Diagnostic Sensitivity of PC Activity Testing

**Figure 2:**
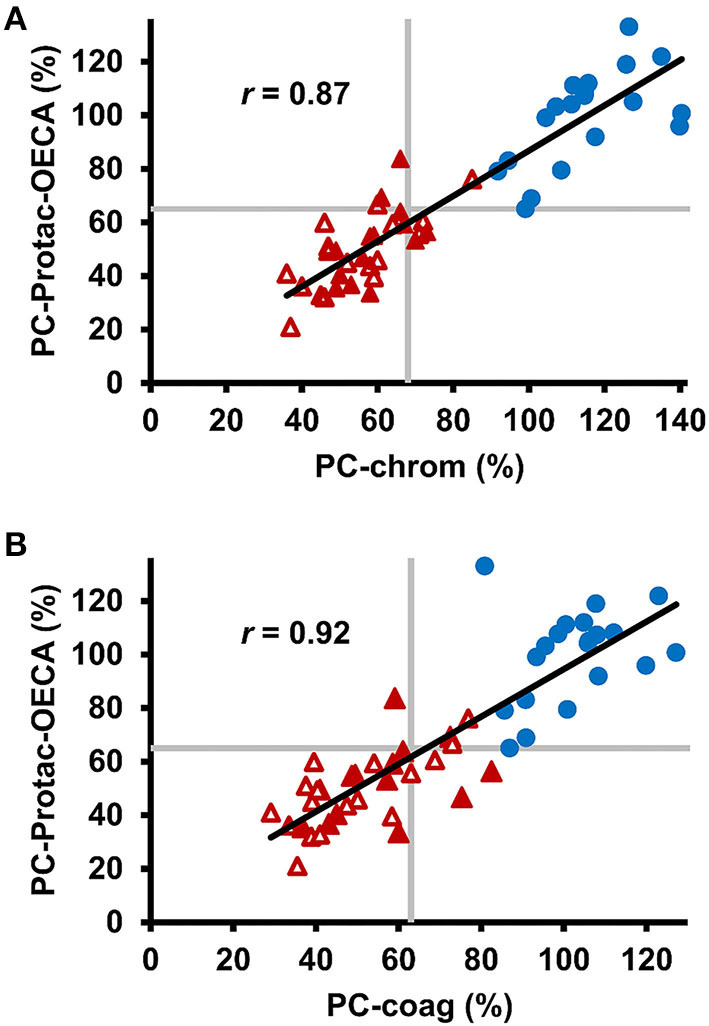
Comparison of chromogenic and clot-based PC activity testing with PC-Protac-OECA in healthy individuals and patients with PROC mutations. **(A)** Correlation between PC-chrom and PC-Protac-OECA in samples from healthy individuals (*n* = 20, blue circles) and patients with PC deficiency causing mutations (*n* = 31, red triangles). Filled and unfilled triangles indicate patients with or without a history of thromboembolic events, respectively. **(B)** Results of PC-coag are plotted against the results of PC-Protac-OECA. Gray lines represent the lower limits of the reference ranges as provided by the manufacturers (PC-chrom and PC-coag), or determined in the healthy controls (PC-Protac-OECA). PC-coag, clot-based PC activity assay; PC-chrom, chromogenic PC activity assay; OECA, oligonucleotide-based enzyme capture assay.

To study, if determination of generated APC through targeting of the basic exosite increases the sensitivity of PC activity testing, the APC-OECA was used to quantify Protac generated APC. Preanalytical stability studies of this PC-Protac-OECA yielded satisfactory results for citrated plasma samples, even when stored at RT for up to 72 h (Supplementary Figure 1). To evaluate the reproducibility of the PC-Protac-OECA one normal and one pathological sample were determined in five simultaneous runs that were repeated on six consecutive days. The calculated coefficients of within-run and between-run variation (CV) of <7% and <10%, respectively, demonstrate the high precision of this approach (Table 2). When testing the 31 patients using this PC-Protac-OECA approach, 27 patients showed a phenotype indicating PC deficiency (Figure 2). Four patients gave false-negative results. Except for one patient (#31, cf. Supplementary Table 1) all these patients were correctly identified as positive in the PC-chrom assay. Vice versa, all patients except for patient #31, who were tested false-negative in the PC-chrom test, were correctly identified as positive in the PC-Protac-OECA (Figure 2A). Comparison of the PC-Protac-OECA results with the PC-coag results showed that 3 of the 7 patients, who showed false-negative results in the PC-coag assay, were identified as true-positive. Three patients (#9, #21, #31) showing false-negative PC-coag results showed also false-negative results in the PC-Protac-OECA (Figure 2B). In addition, one patient (#27), who was correctly identified using the PC-chrom and PC-coag activity assays showed a false-negative result in the PC-Protac-OECA. Based on these results a sensitivity of 87% was calculated for the PC-Protac-OECA.

**Table 2:** Reproducibility of PC-Protac-OECA and PC-TTM-OECA.

**PC input activity, %**	**Mean within-run CV, %** **±** **SD**		**Between-run CV, %**	
**PC-chrom**	**PC-TTM-OECA**	**PC-Protac-OECA**	**PC-TTM-OECA**	**PC-Protac-OECA**
36.9	7.9 ± 5.9	2.6 ± 1.1	15.3	9.5
98.4	4.3 ± 3.0	6.6 ± 2.6	7.1	6.8

*CV, coefficient of variation; PC, Protein C; SD, standard deviation; TTM, thrombin-thrombomodulin*.

### Thrombin-TM-Induced PC Activation Increases the Diagnostic Sensitivity of PC Deficiency Testing

**Figure 3:**
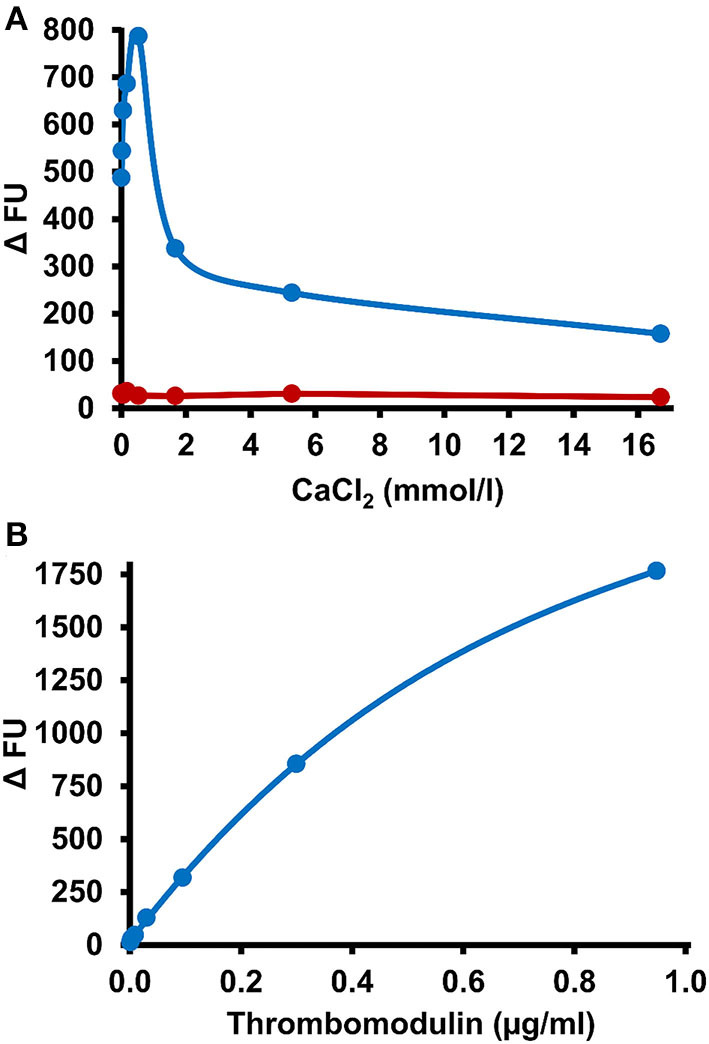
Influence of CaCl_2_ and thrombomodulin on PC activation efficacy. **(A)** A reaction mixture containing CaCl_2_ at the indicated final concentrations and 0.1 μg/ml thrombin was incubated in the absence (red symbols) or presence of thrombomodulin (0.3 μg/ml final concentration, blue symbols) in a 1:10 dilution of citrated plasma in microtiter wells loaded with the APC-binding DNA-aptamer HS02-52G. After 30 min APC formation in the wells was stopped by washing and immobilized APC quantified by measurement of hydrolysis rates of a fluorogenic substrate. **(B)** Indicated final concentrations of thrombomodulin, 0.1 μg/ml thrombin and 0.55 mmol/l CaCl_2_ were applied and assayed as described above. FU, fluorescence units.

To study the effect of the mode of APC generation on the sensitivity of PC deficiency testing, thrombin-TM catalyzed activation of PC was compared with Protac-induced PC activation in the PC-OECA setting. For thrombin-TM-based APC generation, the optimal concentrations of thrombin, TM, and CaCl_2_ were evaluated in initial experiments. The results shown in Figure 3A demonstrate that the V_max_ of APC generation was achieved at 0.55 mmol/l CaCl_2_ while the APC formation rate increased steadily with the TM concentration (Figure 3B). In order to limit the assay costs, we decided not to use higher concentrations of thrombin and TM and therefore applied final concentrations of 0.1 and 0.3 μg/ml (molar ratio of about 1:2), respectively. Thus, for PC-thrombin-TM(TTM)-OECA measurements a solution containing thrombin, TM, and CaCl_2_ (final concentrations of 0.1 μg/ml, 0.3 μg/ml, and 0.55 mmol/l, respectively) was added to plasma samples diluted 1:10 in assay buffer containing 10 μmol/l aprotinin and 1 mg/ml Gly-Pro-Arg-Pro to prevent APC inhibition and fibrin formation. After incubation at 37°C for 30 min, the amount of APC was measured using the APC-OECA as described for the PC-Protac-OECA. The assay performed in this way showed a high reproducibility with a mean within-run CV of <8% and a between-run CV of <16% (Table 2). Pre-analytical stability studies yielded satisfactory results for citrated plasma samples, even when stored at RT for up to 72 h (Supplementary Figure 1).

**Figure 4:**
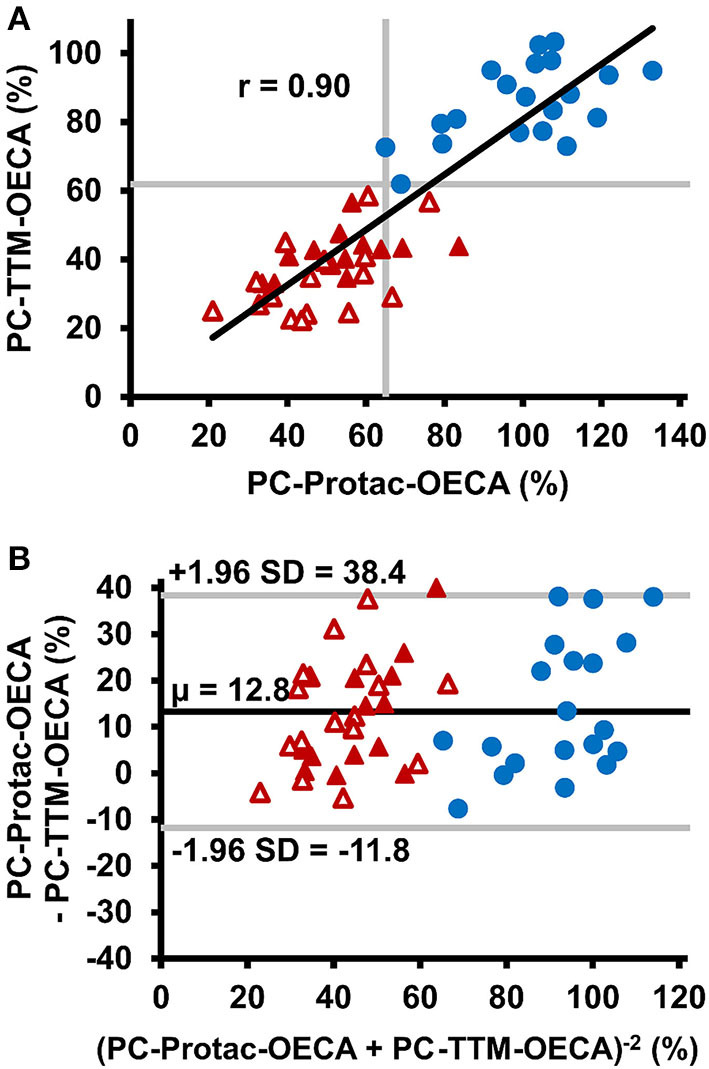
Comparison of thrombin-thrombomodulin activation with Protac activation in PC activity testing. **(A)** Correlation between PC-Protac-OECA and PC-TTM-OECA in samples from healthy individuals (*n* = 20, blue circles) and patients with PC deficiency causing mutations (*n* = 31, red triangles). Filled and unfilled triangles indicate patients with or without a history of thromboembolic events, respectively. Gray lines represent the lower limits of the reference ranges as determined in the healthy individuals. **(B)** Bland-Altman plot of PC-Protac-OECA and PC-TTM-OECA with the black line indicating the mean, and the gray lines indicating the limit of agreement between +1.96 and −1.96 standard deviation. μ, mean; OECA, oligonucleotide-based enzyme capture assay; SD, standard deviation; TTM, thrombin-thrombomodulin.

The PC-TTM-OECA showed no false-negative or false-positive results (Figure 4A). Even patient #31, who gave false-negative results in all other PC tests, was correctly characterized as positive for PC deficiency. The PC-TTM-OECA results correlated well with the PC-Protac-OECA results (Figure 4A), while Bland-Altman analysis revealed a trend toward lower PC levels after thrombin-TM activation (Figure 4B). Additionally, the ratio of PC-TTM-OECA and PC-Protac-OECA results was calculated to assess potential mutation-related differences between the two modes of PC activation. PC-TTM-OECA/PC-Protac-OECA ratios below the range of the controls were calculated for 9 patients, two of them with mutations in exon 6 (#9, #10) and six with mutations in exon 9 (#19, #21, #22, #23, #26, and #27). Among these, #9, #21, and #27 gave false negative results in the PC-Protac-OECA.

## Discussion

Replacing the PC activator Protac by thrombin-TM and subsequent APC detection using an oligonucleotide-based enzyme-capture assay in PC activity testing more precisely identifies a laboratory phenotype as constituted by a PROC gene mutation.

Despite the widespread use of chromogenic PC activity assays as screening tests for inherited PC deficiency, only limited data on the accuracy of these tests, to identify a laboratory phenotype indicative for a mutated PROC gene, are available. Data obtained from survey analyses, such as the analysis of North American specialized coagulation laboratories, suggest a good accuracy of these assays at markedly reduced PC levels, whereas borderline levels are difficult to interpret (14). A major limitation of these studies is the absence of genetic testing as a confirmation or gold standard assay. In the present study we used the PROC genotype as the starting point to test the diagnostic accuracy of routinely used PC tests. With 25 different mutations ranging from single point mutations to large deletions our cohort of patients shows a high genetic diversity and therefore represents a typical cohort of patients with hereditary PC deficiency. However, the spectrum of mutations was neither comprehensive nor representative and might differ from that found in larger populations of VTE patients.

Comparison of chromogenic and clot-based assay results showed a good agreement, but the chromogenic assay measures approximately 10% more PC activity than the clot-based assay. This is supported by previously published data (23). The most likely explanation for this finding is that APC, that is complexed to its inhibitor alpha-2-macroglobulin, retains the ability to cleave small peptide substrates, while the macromolecular substrates FVa and FVIIIa have no access to alpha-2-macroglobulin-complexed APC. This assumption is supported by the close agreement of the PC-chrom results with the results obtained by PC antigen testing.

PC antigen testing by immunological methods such as ELISA is recommended to discriminate between deficiencies of type I and II. Interestingly, PC antigen levels below the reference range were measured in 29 of the 31 patients indicating that even point mutations result in lower detectable plasma levels of PC and suggesting a good diagnostic sensitivity of PC antigen testing. However, this good diagnostic sensitivity is limited by the relatively high rate of false-negative results in patients with type II deficiency, if PC antigen testing was used as a single screening assay.

Decreased PC levels were measured in 84% and in 77% of patients with mutation when using the chromogenic and the clot-based PC activity assays, respectively, indicating that no single test achieves a 100% sensitivity for inherited PC deficiency. The diagnostic sensitivity can be increased to 87% when both tests are used in parallel. However, a small subset of four patients was diagnosed as false-negative by both tests. Moreover, since assay results of the clot-based assay are influenced by a variety of interfering factors such as increased levels of factors V and VIII, and the factor V Leiden mutation, the combination of both assays in routine testing will enhance the rate of false-positive results.

While active site docking is the major step in peptide substrate cleavage, binding of the protein substrates FVa and FVIIIa to APC involves extended surface areas such as the basic exosite of APC (24). These structural differences explain the improved sensitivity of clot-based PC activity tests in the detection of mutations, that affect protein structures outside the catalytic triad. This advantage of clot-based PC activity tests is outweighed by limitations caused through interferences that result in underestimation of PC levels. Another disadvantage of clot-based PC activity testing is its sensitivity to coagulation inhibitors such as heparins, direct acting oral anticoagulants and phospholipid antibodies, that result in overestimation of PC levels (23). Attempting to overcome these limitations, we used the APC-OECA for quantification of generated APC. The basic configuration of this assay is an enzyme capture assay, in which the basic exosite targeting aptamer HS02-52G is used to immobilize APC. HS02-52G bound APC is subsequently determined through hydrolysis of a peptide substrate. Hence, the assay results reflect the integrity of the basic exosite of APC without being influenced by the above-mentioned factors. Evidence, that the OECA-based detection of generated APC mimics that of clot-based detection, comes from the observation that plasma levels of PC were on an average of 12% lower when compared to the chromogenic assay.

The type of PC activation is an additional factor that might influence the diagnostic accuracy of functional PC deficiency testing. This is suggested by a previous study in which a fusion protein of thrombin and the EGF465 domain of TM was used as PC activator and showed a better performance in comparison to Protac in the measurement of normal and PC depleted plasma (25). This approach, however, has not yet been applied in plasma from patients with PROC mutations. Using thrombin-TM as PC activator all plasma samples within our PC deficient patient cohort were correctly identified as true-positive. To further investigate the difference between Protac and thrombin-TM induced PC activation we calculated the ratio between Protac and thrombin-TM-based assay results. A significantly decreased PC-TTM-OECA/PC-Protac-OECA ratio was calculated in patients #9 and #10 with mutations in exon 6, in six patients (#19, #21, #22, #23, #26, and #27) with mutations in exon 9, and in patient #29, who had previously undescribed nucleotide changes in non-coding regions of the PROC gene. Exon 6 encodes the C-terminal of two epidermal growth factor (EGF) regions (2). Mutations in this region have been shown to increase the Ca^2+^ concentration required for PC activation by thrombin-TM (26). As the CaCl_2_ concentration in the PC-TTM-OECA was optimized for activation of wild-type PC, a reduced affinity to calcium could possibly explain the comparably lower PC activity of patients #9 and #10 in the TM-dependent assay. Most PROC mutations associated with a low PC-TTM-OECA/PC-Protac-OECA ratio were located within exon 9, which encodes parts of the anticoagulant serine protease domain of PC. This finding suggests that the molecular regions of APC involved in Protac and thrombin-TM interaction only partially overlap.

Except for severe PC deficiency various studies have shown a poor correlation between the residual PC activity and the thrombotic risk (16, 17). This can be explained by the following hypotheses: (1) there are other confounding factors outside the plasma levels of PC, and (2) the PC activity tests do not accurately reflect the biological activity of PC. The finding that patients with an identical genotype differ in their prothrombogenic potential supports the first assumption.

The data presented here support the conclusion that most of PC deficient patients can be identified by using Protac-based PC activity assays. However, a small subset of patients is diagnosed false-negative. Those patients can be identified by replacing the PC activator Protac with thrombin-TM-activation and subsequently detecting APC using an oligonucleotide-based enzyme capture assay utilizing a basic exosite-targeting aptamer. Further studies in larger samples are warranted to assess, if yet unknown PC mutants can be identified using the new methods of PC activity measurement.

## Data Availability Statement

The original contributions presented in the study are included in the article/Supplementary Material, further inquiries can be directed to the corresponding author/s.

## Ethics Statement

The studies involving human participants were reviewed and approved by Institutional Review Board and Ethics committee of the Medical Faculty of the University of Bonn. The patients/participants provided their written informed consent to participate in this study.

## Author Contributions

SR, HR, and BP contributed to the design of the manuscript. SR and HR collected clinical data. SR and JW performed laboratory analysis. HR performed statistical analysis. SR, HR, JW, JM, AP, JO, and BP critically reviewed the manuscript. JM, AP, and JO contributed to the concept and design of the manuscript and revised the intellectual content. All authors contributed to the article and approved the submitted version.

## Conflict of Interest

The authors declare that the research was conducted in the absence of any commercial or financial relationships that could be construed as a potential conflict of interest.

## Publisher's Note

All claims expressed in this article are solely those of the authors and do not necessarily represent those of their affiliated organizations, or those of the publisher, the editors and the reviewers. Any product that may be evaluated in this article, or claim that may be made by its manufacturer, is not guaranteed or endorsed by the publisher.
