# Supplementary material for: PC Deficiency Testing: Thrombin-Thrombomodulin as PC Activator and Aptamer-Based Enzyme Capturing Increase Diagnostic Accuracy
Source: Front Cardiovasc Med. 2021 Oct 11;8:755281. doi: 10.3389/fcvm.2021.755281 (PMC8542722; doi:10.3389/fcvm.2021.755281)
Supplement: Supplementary file 1 [file Data_Sheet_1.PDF]

Supplementary Material to:

PC Deficiency Testing: Thrombin-Thrombomodulin as PC Activator and Aptamer-based Enzyme Capturing Increase Diagnostic Accuracy

Supplementary Figure

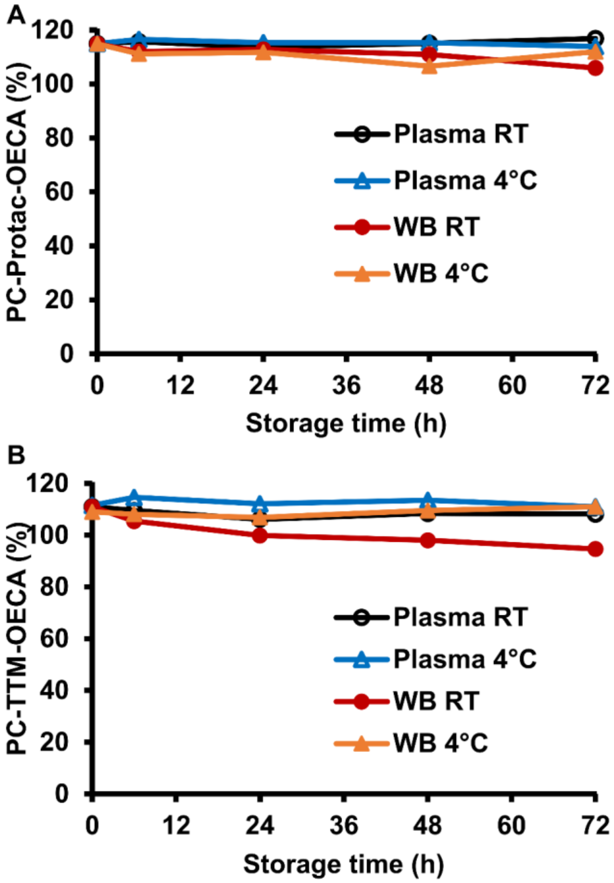

Supplementary Figure 1. Influence of sample storage conditions on **(A)** PC-Protac-OECA and **(B)** PC-TTM-OECA measurement. Citrated whole blood or plasma was stored at room temperature or at 4°C. At the indicated time points aliquots were taken and stored at -40°C before measurement of PC-Protac-OECA and PC-TTM-OECA. WB, whole blood; OECA, oligonucleotide-based enzyme capture assay; RT, room temperature; TTM, thrombin-thrombomodulin.

Supplementary Tables

Supplementary Table 1. Routine PC assay results in patients with inherited PC deficiency

No.	Clinical phenotype	PC-chrom, %	PC-coag, %	PC antigen, %	Type of mutation	Nucleotide exchange
1	asymptomatic	46	39	37	Asp>Asn	c.[262G>A];[=]
2	DVT+PE	56	75	42	Gly>Cys	c.[373G>T];[=]
3	DVT+PE	67	59	45	Gly>Cys	c.[373G>T];[=]
4	asymptomatic	40	34	33	Nonsense	c.[400G>T];[=]
5	DVT	70	57	47	Splice site	c.[400+2T>C];[=]
6	asymptomatic	47	40	38	Nonsense	c.[422C>A];[=]
7	asymptomatic	59	58	56	Gly>Ser	c.[433G>A];[=]
8	DVT	58	49	49	Gly>Ser	c.[433G>A];[=]
9	recurrent DVT	61	73	47	Asp>Val	c.[512A>T];[=]
10	asymptomatic	36	29	29	Nonsense	c.[520C>T];[=]
11	asymptomatic	46	40	36	Nonsense	c.[520C>T];[=]
12	DVT+PE	49	37	33	Large deletion	Exon 6-7
13	recurrent DVT	73	83	59	Arg>Trp	c.[631C>T];[=]
14	DVT	50	45	41	Arg>Trp	c.[658C>T];[=]
15	asymptomatic	37	36	29	Ile>Thr	c.[728T>C];[=]
16	recurrent DVT	53	43	41	Large deletion	Exon 8-9
17	asymptomatic	60	50	44	Arg>Cys	c.[814C>T];[=]
18	DVT+PE	58	60	50	Arg>His	c.[815G>A];[=]
19	asymptomatic	64	54	50	Leu>Ile	c.[866C>T];[=]
20	recurrent DVT	49	41	45	Asn>Asp	c.[892A>G];[=]
21	asymptomatic	60	73	49	Ala>Thr	c.[925G>A];[=]

22	asymptomatic	52	39	48	Ser>Leu	c.[935C>T];[=]
23	asymptomatic	58	48	40	Ser>Leu	c.[935C>T];[=]
24	asymptomatic	47	38	44	Arg>His	c.[983G>A];[=]
25	asymptomatic	45	41	33	Gly>Asp	c.[1028G>A];[=]
26	asymptomatic	71	63	58	Gly>Ser	c.[1234G>A];[=]
27	DVT	66	59	53	Nonsense	c.[1332G>A];[=]
28	recurrent DVT+PE	66	61	45	Nucleotide variation	c.[-41A>T]; [535-42G>A]
29	recurrent DVT	59	50	43	Nucleotide variation	c.[-41A>T]; [535-42G>A]
30	asymptomatic	72	69	66	Splice site	c.[-26C>T];[=]
31	asymptomatic	85	77	66	Splice site	c.[-26C>T];[=]

DVT, deep vein thrombosis; PC, protein C; PC-coag, clot-based PC activity assay; PC-chrom, chromogenic PC activity assay; PE, pulmonary embolism.
